# A Case of Clitoral Hypertrophy of Unknown Origin

**DOI:** 10.1155/2018/7865832

**Published:** 2018-11-01

**Authors:** Tetsuya Okaneya, Kiyoshi Onishi, Michio Saze, Kei Iwakura, Hiroko Sakuma

**Affiliations:** ^1^Department of Plastic and Reconstructive Surgery, Toho University Omori Medical Center, Ota-ku, Tokyo, Japan; ^2^Department of Plastic and Reconstructive Surgery, Hoshi General Hospital, Koriyama-shi, Fukushima, Japan; ^3^Department of Pediatrics, Hoshi General Hospital, Koriyama-shi, Fukushima, Japan

## Abstract

Clitoral hypertrophy is caused by disorders of sex development and it is observed from birth in most cases. We encountered a patient in whom normal morphology at birth may have acquired deformity and hypertrophy. The patient was a 10-year-old girl with a chief complaint of pudendal deformity. The clitoral hood was enlarged and the clitoris size was 8 x 5 mm on the first examination. Various tests were performed. Sex chromosome or hormonal abnormalities and tumorous lesions were not detected, and the ovaries, uterus, and vagina were normal, indicating that disorders of mullerian development were negative. In surgery, reconstruction of the vulva was performed following the Marberger method. The present case may have been a very rare case of acquired hypertrophy of unknown origin.

## 1. Introduction

Clitoral hypertrophy is caused by disorders of sex development and it is observed from birth in most cases. We encountered a patient in whom normal morphology of the clitoris at birth may have acquired deformity and hypertrophy. We report a case with some discussion.

## 2. Case Presentation

**Figure 1:**
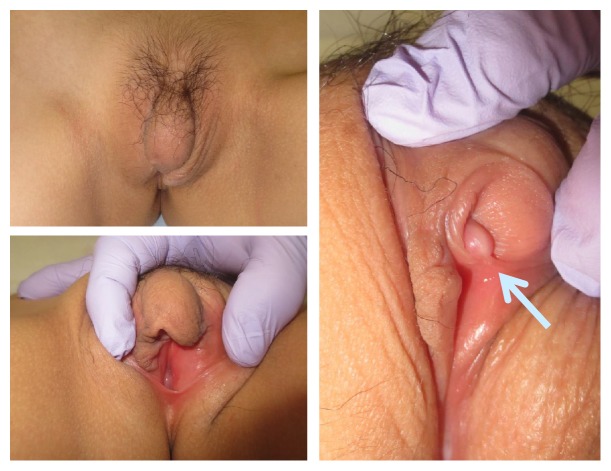
On first examination, the clitoral hood was enlarged and the appearance was similar to the vulva in children with congenital adrenal hyperplasia. The clitoris size (arrow) was 8 x 5 mm.

A 10-year-old girl visited our affiliated hospital, complaining of pudendal deformity. The patient was born at 39 weeks of gestation by normal delivery as the second child, and the birth weight was 3,144 g. There was no disorder in the course of pregnancy in her mother. Intake of androgenic medicine or the disorders of ovaries and uterus was not observed by a periodic medical examination. No pudendal deformity was clearly observed at birth, but lateral asymmetry of the pudendal region was noticed at about 4 years old. A child care worker pointed out that she pressed her heel to the crotch while sitting on her folded legs. After 5 years old, her mother confirmed that her clitoral hood clearly hypertrophied. After entering primary school, a teacher in charge pointed out that she pressed her crotch to a chair or bar, and the mother told her to stop it, but she repeated this behavior every day. When she strongly wanted to be absent from an overnight school trip at 9 years old, the mother brought her to the Pediatric Department. Various tests were performed suspecting disorders of sex development (DSD). On the first visit, the height was 132 cm and the body weight was 26 kg. The clitoral hood was enlarged. The appearance was similar to the vulva in children with congenital adrenal hyperplasia, and the clitoris size was 8 x 5 mm. Labial fusion or adhesion was not detected, and the urinary tract and vagina were open at the normal positions. No masculinization, such as acne and polytrichosis, was noted (Figure 1). Intake of androgenic medicine or the disorders of prepuce was not observed. In the blood test, the sex chromosome was 46, XX. The blood count, blood chemistry, and hormonal test were normal (Table 1). On abdominal ultrasonography, the uterus and ovaries were present. Abdominal CT and MRI examinations showed no tumorous lesion.

**Figure 2:**
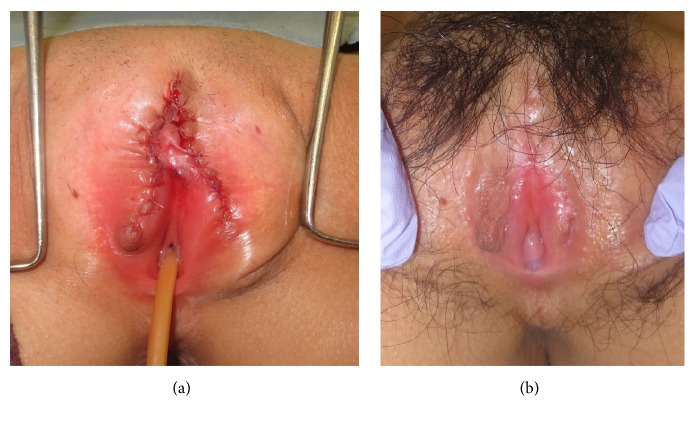
(a) Vulva reconstruction was performed following the Marberger method at completion of surgery. (b) 10 months after surgery.

Based on the above examination and test findings, DSD was considered negative. The patient was diagnosed with clitoral hood enlargement and referred to our department to undergo clitoral hood reduction. For surgery, a longitudinal incision was designed for the dorsal side in order to resect the clitoral hood by cut and try. In the clitoral region, the clitoral hood and corpus cavernosum were dissected through an inverted V-shape incision. The volume of the exposed corpus cavernosum clitoridis was reduced while conserving the neurovascular bundle, following the Marberger method [1]. The clitoral hood was resected into a triangle shape and used for labial formation. On histopathological examination, lymphedema and venous tasis in a grade consistent with the influence of foreskin excision were observed. No abnormality was noted in the corpus cavernosum. Her postoperative course was uneventful. As of 10 months after surgery, favorable improvement of the appearance was noted (Figure 2).

## 3. Discussion

Diseases causing clitoral hypertrophy include congenital adrenal hyperplasia, true hermaphroditism, mixed gonadal dysgenesis, and congenital idiopathic clitoral hypertrophy, and the 3 former diseases account for 95% of cases. All these are congenital and deformity is present since birth. Clitoral hypertrophy associated with these diseases is severe in many cases [2], and many cases have been reported in the gynecology and urology fields [3–5].

In contrast, no surgical treatment of idiopathic clitoral hypertrophy and nonprogressive simple female hermaphroditism with relatively mild hypertrophy without accompanying complication has been reported [6]. It is rarely described even in the field of esthetic surgery. This may be due to the following facts: since the clitoris is the only organ involved only in sexual sensation and it is not directly related to practical problems, such as reproductive and urinary function unlike the penis, its abnormality is likely to be overlooked [7], and abnormality is not readily noticed due to the absence of a reference for comparison because it does not exist in a pair, unlike the labia.

Although clitoral hypertrophy is not a severe disease with regard to the life prognosis, it has a significant influence on patients and their families and impairs the QOL of females. The clitoris is present at the apex in the anterior vaginal vestibule, and it is esthetically problematic even in cases with mild hypertrophy. It caused dyspareunia in some reported cases [7, 8]. Since the morphology of the external genital organs is significantly important for establishing sexual identity in infancy and the disease markedly impairs acquisition of healthy sociability of patients, latent needs for clitoroplasty may be high [9].

In our patient, pudendal abnormality was not clearly noted at birth but deformity occurred with growth. Sex chromosome was 46, XX, no abnormality was detected in hormones on blood testing, tumorous lesions were not detected on imaging, and no anatomic abnormality was observed in the uterus, ovary, vagina, urinary meatus, or vaginal opening. Behavior of pressing the crotch to a chair or bar was frequently noted since infancy, but it was unlikely that this chronic stimulation caused hypertrophy.

Generally, clitoral hypertrophy is defined as a clitoris with a transverse diameter of 7 mm or larger [10]. The size was 8 x 5 mm at 10 years old in this patient, not meeting this criterion, but the appearance clearly suggested clitoral hypertrophy. There may be many cases in which hypertrophy is mild and patients and their families hesitate to visit a hospital because the affected region is the external genital organ even though they have suspicions or feel that something is wrong about its morphological abnormality. It may be necessary to accumulate case reports describing the postoperative sexual function in addition to those on the surgical procedure.

## Figures and Tables

**Table 1:** Blood test findings.

Test item	Result	Normal value	
PRA	5	0.3~5.4	[ng/ml/h]
Aldosterone	198	35.7~240.0	[pg/ml]
Testosterone	10.3	0.8~56.9	[ng/ml]
11-DOF	0.49	~0.6	[ng/ml]
Estradiol	<=5	~20.0	[pg/ml]
LH (CLIA)	<0.1	0.01~0.09	[mIU/ml]
FSH (CLIA)	1.7	0.54~2.47	[mIU/ml]
Cortisol (CLIA)	6.6	~21.1	[*μ*g/dl]
ACTH (ECLIA)	12.2	~55.7	[pg/ml]

PRA: plasma renin activity; 11-DOF: 11-deoxy cortisol; LH: luteinizing hormone; FSH: follicle-stimulating hormone; ACTH: adrenocorticotropic hormone.
